# Clinical and mechanistic relevance of high-dimensionality analysis of the paediatric sepsis immunome

**DOI:** 10.3389/fimmu.2025.1569096

**Published:** 2025-05-13

**Authors:** Dandan Pi, Judith Ju Ming Wong, Katherine Nay Yaung, Nicholas Kim Huat Khoo, Su Li Poh, Martin Wasser, Pavanish Kumar, Thaschawee Arkachaisri, Feng Xu, Herng Lee Tan, Yee Hui Mok, Joo Guan Yeo, Salvatore Albani

**Affiliations:** ^1^ Department of Paediatric Intensive Care Unit, Children’s Hospital of Chongqing Medical University, National Clinical Research Center for Child Health and Disorders, Ministry of Education Key Laboratory of Child Development and Disorders, Chongqing, China; ^2^ China International Science and Technology Cooperation Base of Child Development and Critical Disorders, Chongqing, China; ^3^ Chongqing Key Laboratory of Pediatric Metabolism and Inflammatory Diseases, Chongqing, China; ^4^ Translational Immunology Institute, SingHealth Duke-NUS Academic Medical Centre, Singapore, Singapore; ^5^ Children’s Intensive Care Unit, Department of Pediatric Subspecialties, KK Women’s and Children’s Hospital, Singapore, Singapore; ^6^ Paediatrics Academic Clinical Programme, SingHealth Duke-NUS Academic Medical Centre, Singapore, Singapore; ^7^ Rheumatology and Immunology Service, Department of Pediatric Subspecialties, KK Women’s and Children’s Hospital, Singapore, Singapore; ^8^ Respiratory Therapy Service, Division of Allied Health Specialties, KK Women’s and Children’s Hospital, Singapore, Singapore

**Keywords:** sepsis, severe sepsis, septic shock, pediatric intensive care units, monocytes, Th17 cells, immunology

## Abstract

**Background:**

By employing a high-dimensionality approach, this study aims to identify mechanistically relevant cellular immune signatures that predict poor outcomes.

**Methods:**

This prospective study recruited 39 children with sepsis admitted to the intensive care unit and 19 healthy age-matched children. Peripheral blood mononuclear cells were studied with mass cytometry. Unique cell subsets were identified in the paediatric sepsis immunome and depicted with t-distributed stochastic neighbour embedding (tSNE) plots. Network analysis was performed to quantify interactions between immune subsets. Enriched immune subsets were included in a model for distinguishing sepsis and validated by flow cytometry in an independent cohort.

**Results:**

The median (interquartile range) age and paediatric sequential organ failure assessment (pSOFA) score in this cohort was 5.6(2.0, 11.3) years and 6.6 (IQR: 2.5, 10.1), respectively. High-dimensionality analyses of the immunome in sepsis revealed a loss of coordinated communication between immune subsets, particularly a loss of regulatory/inhibitory interaction between cell types, fewer interactions between cell subsets, and fewer negatively correlated edges than controls. Four independent immune subsets (CD45RA^−^CX3CR1^+^CTLA4^+^CD4^+^ T cells, CD45RA^−^17A^+^CD4^+^ T cells CD15^+^CD14^+^ monocytes, and Ki67^+^ B cells) were increased in sepsis and provide a predictive model for diagnosis with area under the receiver operating characteristic curve, AUC 0.90 (95% confidence interval, CI 0.82–0.98) in the discovery cohort and AUC 0.94 (95% CI 0.83–1.00) in the validation cohort.

**Conclusion:**

The sepsis immunome is deranged with loss of regulatory/inhibitory interactions. Four immune subsets increased in sepsis could be used in a model for diagnosis and prediction of poor outcomes.

## Highlights

- What is already known in this topic. Considered a key healthcare priority by the World Health Organisation, sepsis is a major contributor to global morbidity and mortality with the burden of disease being highest in children and neonates. Nonetheless, paediatric sepsis pathobiology is poorly elucidated and there is no gold standard diagnostic test. As a result, it remains a challenge in clinical practice to differentiate paediatric sepsis from non-infectious insults leading to both under-recognition, overtreatment, and poor outcomes.
- What this study adds. This study adopts a high-dimensionality approach using mass cytometry and leverages on machine learning techniques to characterise the diverse pathophysiological mechanisms in paediatric sepsis. The immune derangement in paediatric sepsis involved all the major immune lineages (CD4^+^ T cell, CD8^+^ T cell, B cell, myeloid, and NK cells) and was characterised by loss of physiological interactions between immune cell subsets—in particular, there was loss of the normal regulatory/inhibitory interactions between cell types. Four pathogenic cell subsets were identified which had good–excellent discriminative ability to distinguish sepsis from healthy.
- How this study might affect research, practice or policy. Paediatric sepsis is characterised by the loss of the normal regulatory/inhibitory interactions between immune cell types. The immune cell subset-based prediction model developed in this study could be used to identify children with sepsis and those with poor outcomes.

## Introduction

Sepsis, identified as a key healthcare priority by the World Health Organisation (WHO), is a major contributor to global morbidity and mortality (1). The global burden of sepsis is highest in children and neonates, with 48 sepsis cases in children per 100,000 person-years and 2,202 per 100,000 live births (2). The corresponding mortality is estimated to range from 1%–5% and 11%–19% in children and neonates, respectively (2). In paediatric severe sepsis and septic shock, mortality can even be as high as 20% in developed countries and 30% in developing countries (3). In addition to the need to advance general medical care in developing countries, a greater understanding of the pathogenic mechanisms underlying paediatric sepsis is required to further lower the global mortality rate in this disease by more targeted strategies (4).

Sepsis is a syndrome including a still-uncertain pathobiology, although activation of both pro- and anti-inflammatory responses is acknowledged, and with no gold standard diagnostic test (5). The current adult and paediatric sepsis definitions consist of clinical and non-specific laboratory criteria (5–7). This may be due to the conventional oligo-dimensional and reductionist approach, which is inadequate to address the mechanistic complexity of sepsis (e.g., examining serum procalcitonin, presepsin, or neutrophil CD64 in isolation) (8). As a result, in clinical practice, it remains a challenge to differentiate sepsis from non-infectious insults and this leads to both under-recognition and overtreatment (9, 10). Another unmet need relates to the inadequacy of existing clinical instruments, such as the Pediatric Index of Mortality 3 (PIM-3) (11) and Pediatric Logistic Organ Dysfunction 2 score (PELOD-2) (12), to predict the likelihood of a poor prognosis, including mortality.

To unravel the complexity of paediatric sepsis, we adopted a high-dimensionality approach using mass cytometry to characterise the circulatory immunome for mechanistically relevant expression of cytokines, chemokines, checkpoint inhibitors, and co-stimulatory receptors. This approach capitalises on machine learning techniques, which apply unsupervised learning to discover hidden patterns in high-dimensional datasets, thus enabling the characterisation of diverse and novel pathophysiological mechanisms in paediatric sepsis (9). In this study, we aim to identify the immune derangements in paediatric sepsis with mass cytometry and depict the dysfunction with a systems biology approach using network analysis. We hypothesised that the paediatric sepsis immunome is typified by multiple immune cellular changes contributing to a perturbed immune network. We also sought to explore the clinical relevance of immune signatures identified by our approach as both support for diagnosis and predictors of clinical fate.

## Materials and methods

### Study design

This study included 93 paediatric subjects: 59 with sepsis admitted to the paediatric intensive care unit (PICU) and 34 healthy age-matched children. This was divided into a discovery cohort of 39 sepsis and 19 healthy children, and a validation cohort of 20 sepsis and 15 healthy children.

A high-dimensionality unsupervised approach using mass cytometry was used to characterise the circulatory immunome in the discovery cohort. Enriched cell subsets provided information on pathophysiological mechanisms and were used as diagnostic and prognostic markers in paediatric sepsis. Flow cytometry was used to confirm the enrichment of identified cell subsets and their performance in the prediction model in the independent validation cohort.

As we aimed to identify the common, overarching immune response in sepsis regardless of the inciting infective agents, we included all children who fulfilled the definition of sepsis irrespective of the underlying infective source and agent. Sepsis was defined as an acute rise in the paediatric sequential organ failure assessment (pSOFA) score ≥2 points in the setting of proven or suspected infection (6). All subjects also fulfilled the International Pediatric Sepsis Consensus Conference (IPSCC) definition for sepsis (13). Severe sepsis and septic shock were defined according to the IPSCC criteria and PICU mortality referred to death prior to discharge from the PICU. Patients with known immunodeficiency or on immunosuppressants were excluded. Sepsis management in the unit was based on the Surviving Sepsis Campaign recommendations (14, 15). Ethical approval from the SingHealth Centralised Institutional Review Board was obtained for this study (CIRB ref. no. 2017–3076 and 2015-2231). Healthy children were recruited among those undergoing elective surgeries with blood obtained at the time of intravenous cannulation prior to the induction of anaesthesia, or postoperatively if the former was not available (CIRB ref no. 2019–2961 and 2015-2231).

### Data extraction

Clinical data for enrolled subjects, including demographic, laboratory, source of infection, and outcome data, were collected. Microbiological tests were ordered at the discretion of the managing physician and may include serology, immunofluorescence, polymerase chain reaction (PCR), agglutination assays, microscopy, isolation/culture, or sequencing techniques. The specimens for these microbiological tests included blood, upper or lower tract aspirates/lavage, pleural/peritoneal/cerebrospinal fluid, urine, stool, and swabs/fluid from sterile sites. Pathogens were reported only if identified within a week of sepsis diagnosis and considered to be the cause of sepsis by the treating physician. Clinical severity scores including the PIM-3 and PELOD-2 were calculated on PICU admission.

### Cell isolation

Blood samples were collected in ethylenediaminetetraacetic acid (EDTA) tubes within 48 h of sepsis diagnosis. Peripheral blood mononuclear cells (PBMCs) were isolated by density centrifugation using Ficoll-Paque Plus (GE Healthcare, UK) and subsequently cryopreserved in foetal calf serum (FCS, Gibco, USA) with 10% (v/v) dimethyl sulfoxide (DMSO, Sigma-Aldrich, UK).

### Mass cytometry

Cryopreserved PBMCs were thawed in Roswell Park Memorial Institute 1640 (RPMI) medium supplemented with 10% (v/v) human serum (Corning, USA) and 1× (v/v) penicillin–streptomycin–glutamine (Gibco, USA). Cells were then resuspended in the same medium and rested for 30 min at 37°C. Subsequently, the cells were harvested and stimulated with phorbol 12-myristate 13 acetate (PMA) at 150 ng/ml and ionomycin at 250 ng/ml. PMA and ionomycin (both from Sigma-Aldrich, UK) stimulation was done for 5 h. PMA-ionomycin bypasses T-cell receptor activation to induce cytokine production enabling it to be detected. Brefeldin A and monensin (eBioscience) were added during the last 3 h of the incubation for blockade of protein transport.

The cells were processed using the standardised EPIC staining protocol as described previously (16). In brief, PBMCs were washed once with cell staining buffer (CSB) (phosphate-buffered saline [PBS] with 4% FCS, 2 mM EDTA, 0.05% sodium azide) and centrifuged at 524 ×*g* for 6 min at 4°C. The supernatant was decanted and cells were stained with cisplatin viability stain (PBS with 10 μM cisplatin) (DVS Sciences, USA) for 5 min on ice. PBMCs were then washed and stained with fluorescein isothiocyanate (FITC) anti-human TCR γ/δ (Invitrogen, USA) at 5 μl and a quadruplet barcode system comprising CD45 antibodies conjugated with Y-89, Cd-106, Cd-113, or Sn-115 (17). After incubation on ice for 20 min, PBMCs were washed three times before they were combined and pelleted in preparation for surface staining with the antibody panel (**Supplementary Table S1**). Mass cytometry combines flow cytometry with mass spectrometry where an antibody–antigen signal is identified by time of flight with heavy metal isotopes [the reader is referred elsewhere for a comprehensive review of mass cytometry technology (18)]. PBMCs were first stained with lanthanide-conjugated surface marker antibodies in room temperature for 15 min in a final reaction volume of 180 μl. After washing twice (initially with CSB and then with 1× PBS), PBMCs were fixed and permeabilised in 1 mL of fixation/permeabilisation buffer (eBioscience, USA) for 45 min on ice. PBMCs were then washed twice with permeabilisation wash buffer (eBioscience, USA) and centrifuged at 840 ×*g* for 6 min. After decanting the supernatant, PBMCs were stained with lanthanide-conjugated intracellular marker antibodies on ice for 45 min in a final reaction volume of 180 μl. PBMCs were subsequently washed once with permeabilisation wash buffer and resuspended in 1× PBS with 1.6% paraformaldehyde (PFA) for 1 day at 4°C prior to data acquisition.

On the day of data acquisition, the cells were pelleted and stained with 500 μl DNA intercalator (DVS Sciences, USA) diluted in 1.6% PFA/1× PBS for 20 min, RT. The cells were then washed twice with CSB and twice with cell acquisition solution (CAS) (Standard BioTools, USA). The pelleted cells were resuspended to a density of 10^6^/ml in UltraPure DNase/RNase-Free Distilled Water with 10% (v/v) EQTM Six Element Calibration Beads (Standard BioTools, USA) in accordance with the manufacturer’s instructions. Data acquisition was then performed using an XT mass cytometer (Standard BioTools, USA).

### Processing of data output from XT mass cytometer

The XT-generated output files were normalised using EQTM Six Element Calibration Beads (19). The live single-cell events and singlets were gated via two steps: first by identifying singlets via a bivariate plot of DNA intercalator versus event length, and next by detecting singlets that are negative for cisplatin as previously described (17). De-barcoding was carried out using a bivariate gating strategy in FlowJo (Version 10.7.1, Becton, Dickinson & Company, USA) and exported for unsupervised analysis.

### Clustering

To identify cell populations in an unsupervised manner, cytometry analysis using self-organising maps (FlowSOM) clustering (20) using a 10 × 10 grid size was applied after random downsampling to 50,000 cell events per subject as previously described (16) (clustering was performed with multiple repeats to ensure stability of cluster phenotype). All clustering and dimensionality reduction operations were preceded by hyperbolic arcsine transformation with a scale factor of 5 (asinh5). Cells were clustered using the FlowSOM algorithm into 100 nodes with subsequent merging based on phenotypic similarity into 47 unique cell subsets. There were 46 subsets obtained after excluding one cluster composed of a mixed cell population from our analysis. Protein expression patterns of clusters were examined using dendrogram heat maps constructed using the “heatmaply” R package.

### Dimensionality reduction

Principal component analysis (PCA) was used to extract and visualise the dominant patterns in the matrix prior to tSNE dimensionality reduction (21). Non-linear dimensionality reduction was performed using t-distributed stochastic neighbour embedding (tSNE) (22) to visualise multidimensional expression landscapes in two dimensions (2D). “Relatedness” among different clusters was visualised after embedding FlowSOM clustering information onto the 2D tSNE plots.

### Network analysis

The proportion of nodes (immune cell subsets) was calculated for every patient, and the correlation between nodes was calculated for healthy control and paediatric sepsis groups. To construct the network, nodes were connected if they had an absolute correlation coefficient >0.6. The network was visualised and analysed using the igraph R package. The correlation network was plotted using a force-directed Fruchterman–Reingold graph layout (23).

### Flow cytometry validation

Significantly increased subsets identified on mass cytometry were validated using flow cytometry in an independent cohort. Two flow cytometry antibody panels (**Supplementary Table S2**) were designed for the validation. The thawing, stimulation, and staining protocol mirrored that of the mass cytometry protocol described above, with the exception that Live/Dead blue dye (Invitrogen) was used for 15 min at room temperature in PBS. Stained samples were then analysed using the LSR Fortessa™ flow cytometer (BD Bioscience). Verification of the FlowSOM clustering frequency (expressed as a percentage of CD45^+^ PBMCs) was performed with bivariate supervised gating in FlowJo (Version 10.7.1, BD, USA) (**Supplementary Figures S1**, **S2**).

### Statistical analysis

Cell subset frequencies were plotted as median with interquartile range (IQR). Chi-squared/Fisher’s exact and Mann–Whitney U and tests were used to compare groups where appropriate. To account for type I error, Bonferroni correction was used when comparing the FlowSOM cell clusters obtained from the mass cytometry data between sepsis and control groups. Acknowledging the process of immune development and maturity continues in early life (16, 24), a sensitivity analysis stratifying children into age >/<1year was performed to determine if any changes in cell subset frequencies differed with age. Correlation between variables was calculated either with Pearson (r) or Spearman (r_s_) correlation coefficients. Significantly increased cell subsets in paediatric sepsis were considered for inclusion in a model to differentiate sepsis from healthy, and to predict severe sepsis, septic shock, and PICU mortality among septic patients. The discriminative ability and performance of these models was assessed by calculating the area under the curve from final receiver operating characteristics curve (AUROC), sensitivity, and specificity. For comparison, AUROC was similarly calculated to determine discriminative ability and performance of clinical scores and routine laboratory markers (e.g., procalcitonin and lactate). Analyses were performed using SPSS, version 23.0 (IBM Corp., NY, USA), and GraphPad Prism V.7 (GraphPad Software, Inc., CA, USA) with statistical significance set at p < 0.05.

## Results

### Baseline characteristics of the study population

The median age and pSOFA score of the paediatric sepsis cohort were 5.6 (IQR: 2.0, 11.3) years and 6.6 (IQR: 2.5, 10.1), respectively (**Table 1**). Most patients had pneumonia/lower respiratory tract infection [42/59 (71.2%)] and 11 (18.6%) had blood culture-confirmed bacteraemia. Multiorgan dysfunction was present in 41/59 (69.5%) and mortality occurred in 8/59 (13.6%) of patients. Clinical characteristics of the PICU survivors and non-survivors are presented in **Supplementary Table S3**. The microbiological data are shown in **Supplementary Table S4**.

**Table 1:** Demographic and clinical characteristics of sepsis and control individuals.

	Sepsis patients (n=59)	Healthy controls (n=34)
Demographics		
Age(years)	5.6(2.0, 11.3)	6.6 (2.5, 10.1)
Age group		
Neonate (<1month)	1 (1.7)	2 (6.1)
Infant (1–12 months)	11 (18.6)	3 (9.1)
Child (1–12 years)	33 (55.9)	25 (75.8)
Adolescent (>12 years)	14 (23.7)	4 (11.8)
Male gender, n(%)	35 (59.3)	28 (84.9)
Laboratory examinations		
White blood count, 10^9^/L	9.5 (5.4, 20.9)	
C-reactive protein, mg/L	119.0 (32.1, 203.0)	
Procalcitonin, ng/mL	6.1 (1.0, 62.0)	
Lactate, mmol/L	1.6 (1.0, 3.1)	
Severity of disease*		
pSOFA	8 (5, 11)	
PIM-3	3.4 (1.6, 6.4)	
PELOD-2	6 (3, 8)	
Sites of infection, n(%)		
Respiratory	42 (71.2)	
Systemic	5 (8.5)	
Gastrointestinal	7 (11.9)	
Central nervous system	2 (3.4)	
Musculoskeletal	2 (3.4)	
Genitourinary	1 (1.7)	
Pathogen, n(%)		
Bacteria	15 (25.4)	
Virus	17 (28.8)	
Negative	19 (32.2)	
Mixed	7 (11.9)	
Multiorgan dysfunction, n(%)	41 (69.5)	
Severe sepsis, n(%)	44 (74.6)	
Septic shock, n(%)	43 (72.9)	
Mortality in PICU, n(%)	8 (13.6)	

^*^Scored at PICU admission. Data are expressed as median (interquartile range) unless otherwise indicated. PELOD-2, paediatric logistic organ dysfunction 2 score; PIM3, paediatric index of mortality 3; pSOFA, paediatric sequential organ failure assessment; PICU, paediatric intensive care unit.

### A high dimensionality approach reveals a multitude of immune cellular derangements and a dysregulated immune network in paediatric sepsis

**Figure 1:**
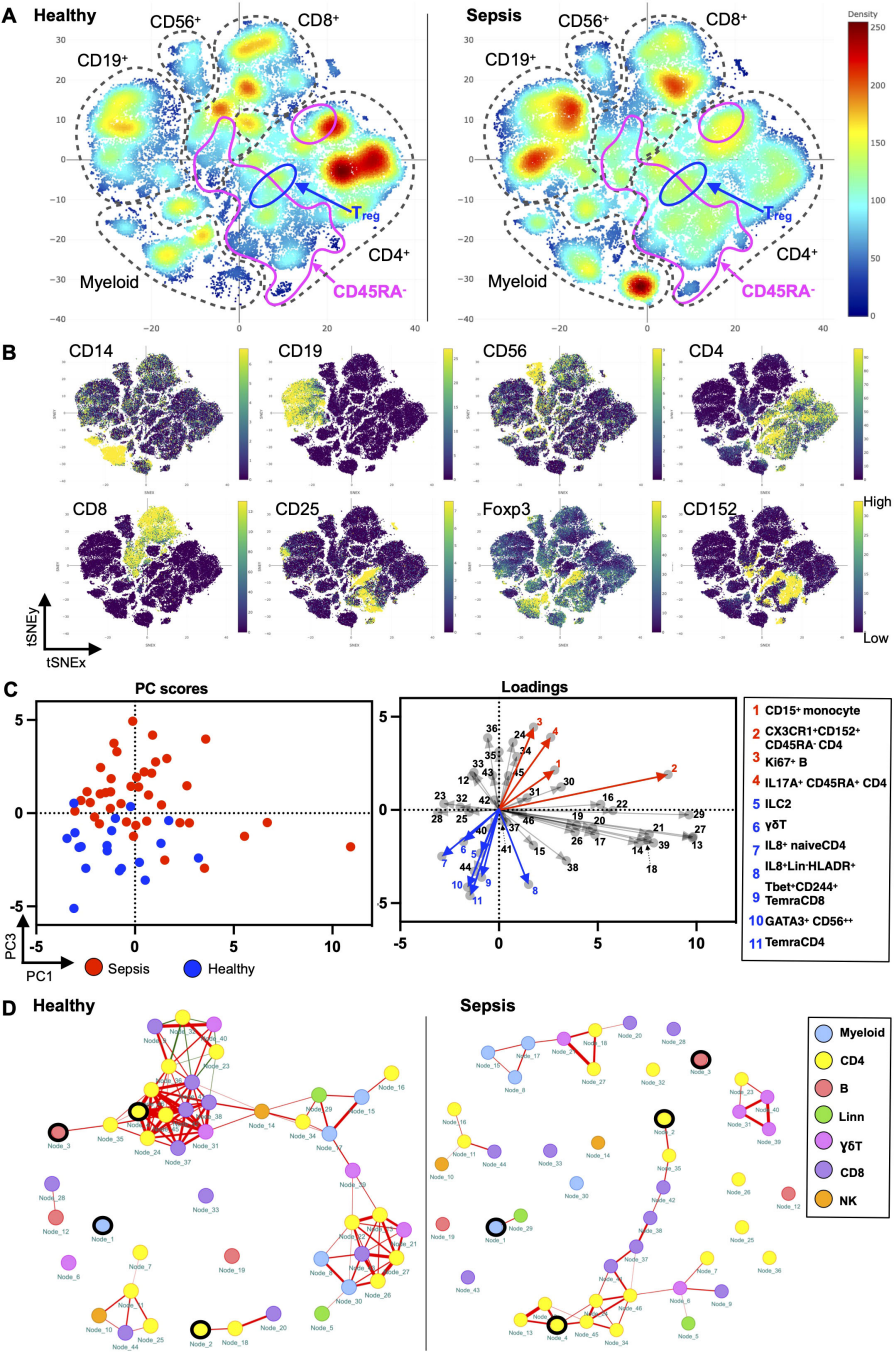
Paediatric sepsis involves derangement in multiple immune cell subsets. **(A)** Density plots after t-SNE dimensionality reduction demonstrate differences between paediatric sepsis and healthy immunomes. Each density plot is derived from the random sampling of 50,000 single events from the concatenated mass cytometry data from 39 paediatric patients with sepsis and 19 healthy donors. The purple solid outlines demarcate the regions of single-cell events that are CD45RA^−^, denoting a memory T-cell phenotype. The T regulatory cell clusters are demarcated with a solid black line. **(B)** t-SNE plots with embedded marker expression included 50,000 randomly sampled single-cell events from the concatenated mass cytometry from subjects with sepsis and healthy donors. The presence of CD25, Foxp3, and CD152 (CTLA4) expression defines the T regulatory cells. **(C)** PCA segregates the subjects with sepsis from the healthy donors. PC1 and PC3 account for 24.5% of the observed variance. The loading plot depicts the differential contribution of the 46 immune cell subsets to PC1 and PC3. The four and seven subsets that were increased and reduced in paediatric sepsis were coloured in red and blue, respectively. **(D)** The paediatric sepsis immune network has a reduced number of intercellular interactions and negatively correlated edges. Red: positive correlation, green: negative correlation. All represented correlations are statistically significant. The phenotypes of the nodes (cell subsets) for **(C, D)** based on their node numbers, are listed in **Supplementary Table S5**. The four increased cell subsets that are increased in sepsis are encircled in black.

To holistically depict the immunome in paediatric sepsis, we compared the circulatory immune profiles of children with sepsis to healthy children with mass cytometry. We observed changes in the immune cell composition within all the major immune lineages (CD4^+^ T cell, CD8^+^ T cell, B cell, myeloid, and NK cells) between the two groups (**Figures 1A, B**).

These immunological changes were able to segregate subjects with sepsis and in health (**Figure 1C**). A plot of principal component (PC) 1 and PC3 showed this separation when it was performed on the 46 cell subsets identified (**Figure 1C**, **Supplementary Table S5)**. The loading plot showed the coefficients of the linear combination of different immune subsets from which the PCs are constructed (**Table 2**). As illustrated, different subsets contributed variably to the clinical dichotomisation observed with PCA. Eleven out of the 46 cell subsets were significantly different between the sepsis and healthy groups and annotated onto the loading plot to depict their contribution to the PCA. The four and seven subsets that were increased and reduced in paediatric sepsis were coloured in red and blue, respectively (**Figure 1C**).

**Table 2:** Subset information for principal component analysis (PCA) loading plot.

Top 10 subsets in PC3 (PC score)	Top 10 subsets in PC1 (PC score)
temraCD4 (−0.670)	IL4^+^TIGIT^+^CX3CR1^+^CXCR3^+^CCR4^+^ naiveCD4 (0.762)
GATA3^+^CD56^++^ (−0.604)	CD160^+^CX3CR1^+^CXCR3^+^CCR4^+^ effector memory CD4 (0.760)
Ki67^+^ B (0.589)	Lineage negative^-^ (0.746)
Lin^-^HLADR^+^IL8^+^ (−0.582)	CD45RA^-^CX3CR1^+^CTLA4^+^CD4^+^ (0.664)
Tbet^+^CD244^+^ temraCD8 (−0.526)	IL8^+^ naïve CD8 (−0.659)
CD45RA^−^IL17A^+^CD4^+^ (0.517)	IL8^+^ naïve CD4 (−0.616)
PD1^+^CD152^+^TIGIT^+^ effector memory CD4 (0.513)	Tbet^+^GB^+^CX3CR1^+^CXCR3^+^ gdT (0.607)
FOXP3^+^CD25^+^CD152^+^PD1^+^TIGIT^+^CCR4^+^ Treg (0.482)	FOXP3^+^CD25^+^CD152^+^ naïve Treg (−0.593)
temraCD8 (−0.480)	Double negative T cell (0.590)
PD1^+^CD152^+^Ki67^+^ central memory CD4 (0.414)	CX3CR1^+^CXCR3^+^ naïve CD4 (0.582)

List of abbreviations can be found in the **Supplementary Material**.

Next, we used network analysis to determine how immune cells interact and coordinate at the system level in sepsis. For this, a correlation network was constructed with the 46 distinct cell subsets (**Figure 1D**; **Supplementary Table S6**). The correlation of the frequency of each node (cell subset), expressed as a percentage of total PBMC, with other nodes was calculated. The correlations among the nodes were used to define the edges (interconnections) between the nodes in the cellular network. The colour of the edge denotes the direction of the correlation (red, positive; green, negative), whereas its thickness indicates the strength of the absolute correlation.

From this analysis, it is evident that the immune cell network of children with sepsis differs from the network constructed with healthy children (**Figure 1D**). There is a reduced number of interactions among immune cell subsets in the sepsis network suggesting a loss of coordinated communication between immune cell subsets in sepsis. The network density, an overall measure of interaction among nodes in the network, is lower (0.040) for paediatric sepsis compared with healthy (0.103) (**Supplementary Table S6**).

The immune cell network showed that the number of negatively correlated edges was 0 in the paediatric sepsis network compared with 8 (7.48% of all edges) in the healthy network. The absence of negatively correlated edges suggests a loss of counterbalancing forces. Notably, the naïve T regulatory cell subset (node 23) and PD1^+^CD152^+^TIGIT^+^ effector memory CD4^+^ T cells (node 36) are among the nodes with negative edges.

Furthermore, the paediatric sepsis network had a higher modularity (0.242) compared with the healthy network (0.002) (**Supplementary Table S6**). The higher modularity in the paediatric sepsis network is attributed to the formation of a restricted communication module between CD4^+^ and CD8^+^ T-cell subsets with each other. Comparatively, the cell subsets in healthy children interact across different cell lineages. The CD4^+^ T-cell subsets involve more pro-inflammatory factors such as TNFα and INFγ (**Figure 1D**; **Supplementary Table S5**). Henceforth, a loss of negatively regulated intercellular connections characterises the paediatric sepsis immunome network.

### Enrichment of CD15^+^CD14^+^ monocytes, CD45RA^−^CX3CR1^+^CTLA4^+^CD4^+^ T cells, and CD45RA^−^17A^+^CD4^+^ T cells is associated with greater disease severity

**Figure 2:**
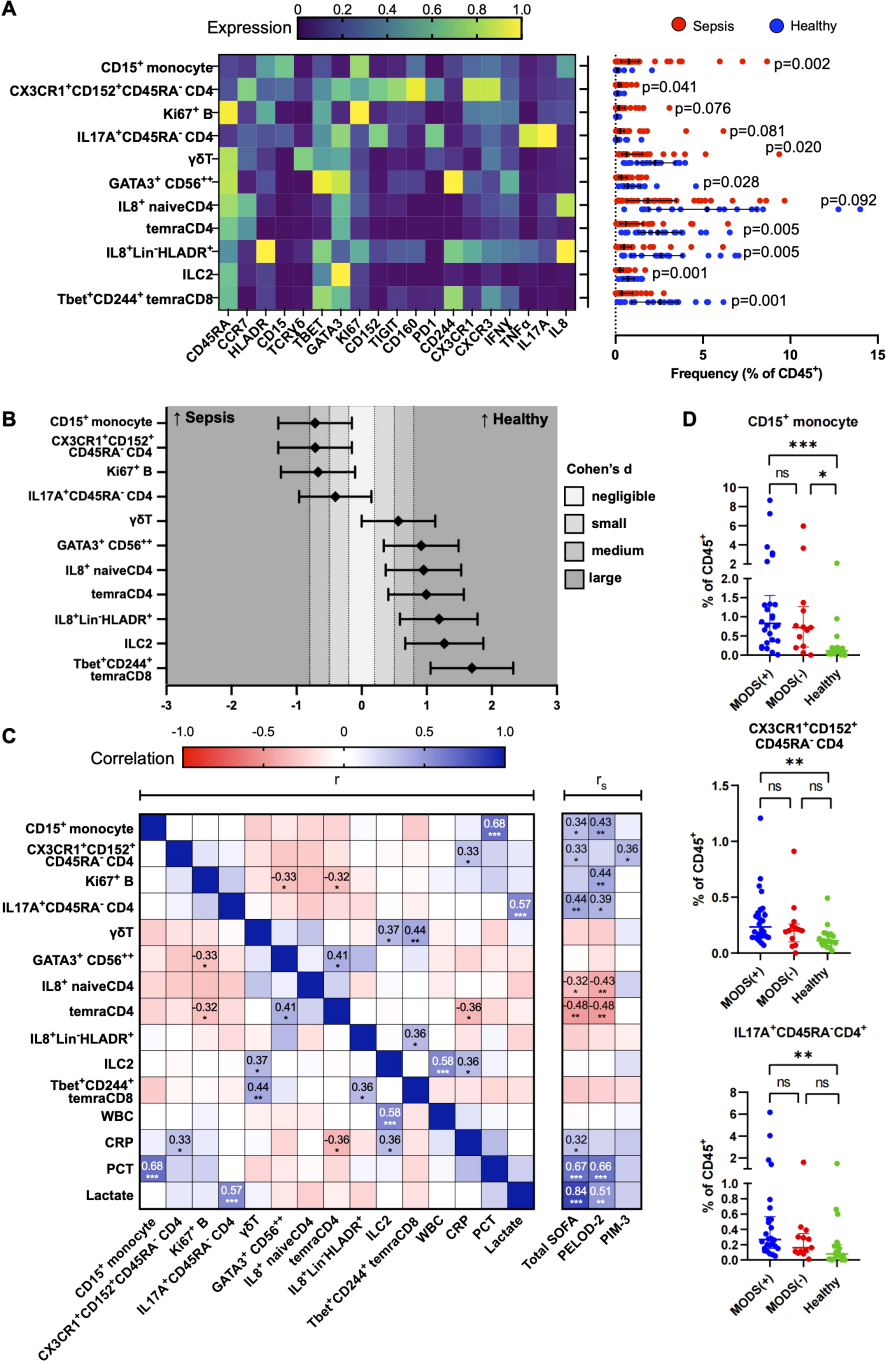
Immune cellular derangements in paediatric sepsis and their correlation with clinical scores. **(A)** Scaled median (arcsine transformed) marker expression profiles (heat maps) of merged FlowSOM-derived cell clusters that are significantly altered in paediatric sepsis and their frequencies depicted as a percentage of CD45^+^ PBMC (n = 39, paediatric sepsis and n = 19, healthy donors). Boxplots indicate median and IQR. Statistical testing: p-values from Mann–Whitney U (two-tailed) test with Bonferroni correction are shown. A cutoff p-value of < 0.1 was used as our threshold for type I error. Stimulated PBMCs (with PMA and ionomycin) were studied. **(B)** Effect size of each significantly deranged cell subset to paediatric sepsis. Cohen’s d statistics (standardised effect size) with 95% confidence intervals are depicted. **(C)** Correlation matrix among significantly deranged cell subsets and conventional laboratory tests and clinical severity scores. Correlation coefficients, r: Pearson, r_S_: Spearman. **(D)** Distribution of immune subsets according to the number of organ dysfunction (groups 2 and 6 only had two patients and were collapsed into the preceding group). Organ dysfunction was defined by the International Consensus Conference on Pediatric Sepsis. WBC, white blood cell; CRP, C-reactive protein; PCT, procalcitonin; SOFA, paediatric sequential organ failure assessment; PELOD-2, paediatric logistic organ dysfunction score-2; PIM-3, paediatric index of mortality 3. *p<0.05, **p<0.01, ***p<0.001.

**Figure 2A** depicts the marker expression heatmap and cell frequencies of the 11 cell subsets that were significantly different between the sepsis and healthy groups after Bonferroni correction. CD15^+^CD14^+^ monocytes, CD45RA^−^17A^+^CD4^+^ T cells, CD45RA^−^CX3CR1^+^CTLA4^+^CD4^+^ T cells, and Ki67^+^ B cells were significantly increased in patients with sepsis, with the CD15^+^CD14^+^ monocytes having the largest normalised effect size in sepsis (**Figure 2B**). The four cell subsets discovered with an unsupervised analysis with FlowSOM clustering were validated with a supervised analysis with traditional bivariate gating (**Supplementary Figures S3A, B**). All except for the Ki67^+^ B cells were significantly different on further validation with fluorescence-based flow cytometry and were included for further downstream analysis for association with clinical severity and outcomes.

To determine if the changes in these 11 cell subsets differed with age, we performed a subgroup analysis by stratifying them into children with age >1 year [74.4% (29/39) in the sepsis group and 73.7% (14/19) in the healthy group] and those ≤1 year of age (**Supplementary Figures S4**, **S5**). The CD15^+^CD14^+^ monocytes and CD45RA^−^CX3CR1^+^CTLA4^+^CD4^+^ T cells were only significantly increased in children with sepsis in those >1 year of age (**Supplementary Figures S4A, B**). Contrastingly, the CD45RA^−^17A^+^CD4^+^ T cells were significantly increased in those with sepsis in both age groups (**Supplementary Figure S4D**). This was not due to greater disease severity in the older children (>1 year old) as the pSOFA and PELOD2 scores were comparable, and the PIM-3 score was, in fact, higher in the ≤1-year group (**Supplementary Figure S4E**). The other seven cell subsets that were significantly decreased in sepsis were also only present in children >1 year of age (**Supplementary Figure S5**).

Next, we evaluated the clinical relevance of these findings with sepsis severity scores and the occurrence of multiorgan dysfunction (**Figures 2C, D**). The increases in the CD15^+^CD14^+^ monocytes, CD45RA^−^17A^+^CD4^+^ T cells, and CD45RA^−^CX3CR1^+^CTLA4^+^CD4^+^ T-cell subsets were independent and not correlated with each other (**Figure 2C**). There was a positive correlation between the frequency of CD15^+^CD14^+^ monocytes with serum procalcitonin (Pearson’s r=0.68, p<0.001), and CD45RA^−^ CD45RA^−^17A^+^CD4^+^ T cells with serum lactate (Pearson’s r=0.57, p<0.001). Although both CD15^+^CD14^+^ monocytes and CD45RA^−^17A^+^CD4^+^ T cells were significantly correlated with the clinical severity scores, pSOFA and PELOD-2, serum procalcitonin, and lactate yielded a stronger correlation with pSOFA and PELOD2, than the identified subsets. There was a progressive increase in the frequency of the CD15^+^CD14^+^ monocytes, CD45RA^−^17A^+^CD4^+^ T cells, and CD45RA^-^CX3CR1^+^CTLA4^+^CD4^+^ T-cell subsets from control to sepsis with organ dysfunction (**Figure 2D**).

### Enrichment of CD15^+^CD14^+^ monocytes, CD45RA^−^CX3CR1^+^CTLA4^+^CD4^+^ T cells, and CD45RA^−^17A^+^CD4^+^ T cells aids diagnosis and predicts mortality

**Figure 3:**
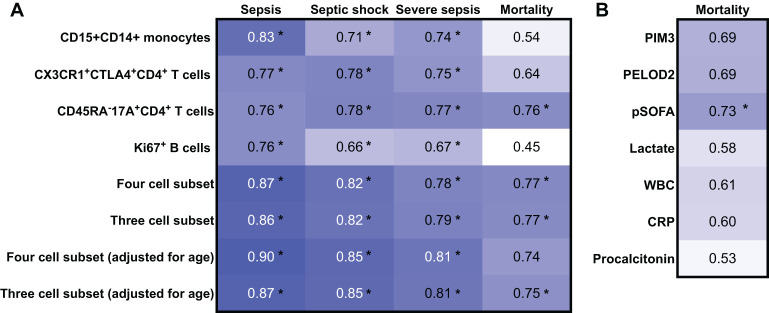
Receiver operating characteristic (ROC) analysis of the discovery cohort for the diagnosis of sepsis, sepsis shock, and severe sepsis and for the prediction of mortality. AUROC values derived from mass cytometry data from the discovery cohort (n = 58). *Indicates that the 95% confidence intervals do not cross 0.5.

ROC analysis was performed to discriminate paediatric sepsis from healthy and to predict septic shock, severe sepsis, and mortality among the discovery cohort (**Figure 3A)**. Individually, higher frequency of CD15+CD14+monocytes [0.83 (95%CI 0.71,0.94)], CD45RA^-^CX3CR1^+^CTLA4^+^CD4^+^ T cells [0.77 (95%CI 0.64,0.90)], CD45RA^−^17A^+^CD4^+^ T cells [0.76 (95% CI 0.61,0.90)], and Ki67^+^ B cells [0.76 (95%CI 0.64, 0.88)] yielded good AUROC for distinguishing sepsis from healthy. When all four subsets were combined and adjusted for age, AUROC improved to 0.90 (95%CI 0.82, 0.98) with a sensitivity of 87.2%, specificity of 79.0%, positive predictive value of 89.5%, and negative predictive value of 75.0%, thus suggesting that the determination of these combined variables may be considered as a diagnostic aid for paediatric sepsis. The AUROC of the four-subset model adjusted for age was acceptable for the diagnosis of severe sepsis and septic shock as well. When reduced to three cell subsets (excluding Ki67^+^ B cells), performance of the model to distinguish sepsis, severe sepsis, and septic shock remained good–excellent.

The same three cell-subset model, adjusted for age, was comparable with the tools currently used in clinical practice to predict mortality [0.75 (95%CI 0.53, 0.98)], such as the PIM-3, PELOD2, and pSOFA score, and better than biomarkers serum C-reactive protein, procalcitonin, and lactate (**Figure 3B**).

To validate these findings, the four cell subsets were identified by a different technique, namely, flow cytometry, and shown to be increased consistently in an independent cohort (**Supplementary Figure S6C**). A representative gating strategy of one sepsis patient and one healthy control is shown in **Supplementary Figures S4A, B**. Repeat ROC analysis with flow cytometry-derived cell frequencies produced consistent results with the four and three cell-subset model adjusted for age yielding good–excellent AUROC to distinguish sepsis from healthy [0.87 (95%CI 0.72, 1.00) and 0.86 (95%CI 0.71, 1.00), respectively] (**Supplementary Figure S7**). As flow cytometry easily allowed determination of cell lineage frequency, the four and three cell-subset information based on cell lineage frequency performed even better to distinguish sepsis from healthy [0.94 (95%CI 0.83, 1.00) and 0.94 (95%CI 0.83, 1.00), respectively].

## Discussion

Given the myriad of triggering microbiological agents and the complexity of the host sepsis response, reductionist approaches based on individual cellular or biochemical markers are unlikely to provide suitable biomarkers for sepsis diagnosis or prognostic stratification. Instead, multivariate approaches have been shown to be more promising, even though the ones available to date are still limited in scope to specific clinical subsets and do not employ technologies which can comprehensively define the architecture of the immune system (25–27). A high-dimensionality approach where the architecture of the immunome is derived holistically at the single-cell proteome level and analysed by machine learning techniques using supervised (linking known variables to outcomes) or unsupervised (to determine hidden patterns of unknown variables to outcomes) algorithms may be the solution to addressing the complex nature of sepsis.

In the discovery cohort, immune derangements in paediatric sepsis were identified using single-cell mass cytometry with a systems biology approach and network analysis. These derangements were characterised by the reduction in the interactions between otherwise normally highly connected immune cell subsets as found in health. In particular, there was a loss of the normal regulatory/inhibitory interactions between cell types. This observation may provide a novel contribution to understanding the pathogenesis of the disease. Indeed, in response to an infective agent, the innate and adaptive immune responses are triggered to defend the host from pathogen invasion (28). Interactions between the innate and adaptive immune elements are crucial for effective clearance of pathogens, and yet this must also be regulated to avoid inadvertent/excessive inflammatory injury (29). In our study, the immune cell network of children with sepsis demonstrated fewer interactions compared with healthy subjects indicating a loss of coordinated communication between immune cell subsets in sepsis. There was an absence of negatively correlated edges suggesting loss of regulatory/inhibitory forces—notably, the T regulatory and PD1 inhibitory CD4^+^ T-cell subsets did not produce regulatory/inhibitory effects as expected, potentially leading to excessive inflammatory injury. Despite evidence of interactions between CD4^+^ and CD8^+^ T-cell subsets, such interactions occurred in restricted communication modules in sepsis. In contrast, interaction between immune cell subsets in healthy control subjects occurred in a balanced fashion across functionally different subsets (including NK cells, myeloid cells, and B cells). Altogether, the concept of centrality and connectivity of cell subsets provides a different pathogenic perspective to the traditional approach of simply measuring the number of cells to explain their functional and pathogenic relevance in the network of a diseased immunome.

Importantly, our approach also identified several immune cell subsets which were significantly different with controls. We combined the four immune cell subsets which were increased for several reasons. They were independently increased (i.e., showing no interdependency and correlation between them); they possessed mechanistically meaningful markers such as CD15 (cell adhesion), CX3CR1 (chemokine receptor), Ki67 (proliferation), and IL17A (pro-inflammatory cytokine); they possessed significant correlations with clinical scores (pSOFA, PIM-3, PELOD-2); and lastly, being increased in the disease state allowed us to conduct further downstream functional studies with sufficient cell numbers with fluorescence activated flow sorting. When combined in a model, higher frequency of these subsets had good-to-excellent discriminative ability to distinguish sepsis from healthy, and fair-to-moderate discriminative ability to predict non-survivorship.

The cell subsets identified are supported by evidence of biological plausibility. Although functional experiments were not part of the aims of this study, previous reports provide context on the mechanistic relevance of these cell subsets. Classical monocytes, part of the innate immune response, comprise the majority of circulating monocytes (80%–90%) and perform several roles in sepsis, including antigen presentation, phagocytosis of pathogens, and production of pro- and anti-inflammatory cytokines (30, 31). The CD15 marker indicates that this subset is likely in the mature monocyte stage of development. Specifically, CD15 functions as a monocyte counter-receptor which engages endothelial cell E-selectin leading to monocyte adhesion, activation, and pro-inflammatory cytokine production (32). A greater abundance of CD15^+^ monocytes may be associated with a greater pro-inflammatory potential. Central memory CD4^+^ T cells play a central immune surveillance role, patrolling lymph nodes and proliferating to produce both effector and effector memory T cells when encountering a familiar antigen (33). CX3CR1 expression marks the most differentiated Th1 effector population and functionally serves as a tissue homing receptor toward the ligand CX3CL1 (fractalkine) produced at sites of infection (34). High systemic levels of CX3CL1, however, are associated with multiorgan dysfunction, impaired pathogen clearance, and mortality (35). Lastly, IL-17-driven inflammation has been established in mediating effects like organ dysfunction (36, 37) and mortality (38, 39) in sepsis.

Altogether, this work corroborates the validity of the high-dimensionality, machine learning-powered approach to understand the diseased immunome in sepsis for its derangements also at the level of network. Our findings, which were validated by the use of an independent technology and independent cohort, provides a tool to aid diagnosis and may be superior to existing clinical scoring systems and clinically available biomarkers in predicting mortality (40). This work would be certainly strengthened by testing in a larger and more diverse cohort of patients including infectious non-sepsis, non-infectious systemic inflammatory response and immunosuppression. Further to this, our study provides pilot data for a diagnostic clinical trial to determine if early measurement of these biomarkers translate to improved/appropriate treatment for sepsis. Moreover, neutrophils which are known to play a significant role in sepsis and organ failure were not considered in our analysis (due to the loss of neutrophils during the cryopreservation process). Finally, our data may also help in proposing further research into therapies employing CX3CR1 antagonists (AZD8797) (41) or humanised monoclonal antibody of IL-17A (ixekizumab) (42, 43) as potential therapeutic targets in sepsis.

## Data Availability

The raw data supporting the conclusions of this article will be made available by the authors, without undue reservation.
