# Supplementary material for: Clinical and mechanistic relevance of high-dimensionality analysis of the paediatric sepsis immunome
Source: Front Immunol. 2025 May 13;16:1569096. doi: 10.3389/fimmu.2025.1569096 (PMC12106532; doi:10.3389/fimmu.2025.1569096)
Supplement: Supplementary file 1 [file SupplementaryFile1.pdf]

Supplementary Material

Title: Clinical and mechanistic relevance of high dimensionality analysis of the paediatric sepsis immunome

Authors: Dandan Pi†, Judith Ju-Ming Wong†, Katherine Nay Yaung, Nicholas Khoo, Su Li Poh, Martin Wasser, Pavanish Kumar, Thaschawee Arkachaisri, Feng Xu, Herng Lee Tan, Yee Hui Mok, Joo Guan Yeo‡, Salvatore Albani‡

(†, ‡ These authors contributed equally to this work)

Contents

		Page
1.	Detailed Methodology	*3-5*
2.	Table S1. Mass Cytometry panel antibodies conjugated metal, clone and vendor information	6-7
3.	Table S2: Flow Cytometry Validation Panel	8
4.	Table S3. Demographic and clinical characteristics of sepsis survivors and non-survivors	9
5.	Table S4: Pathogens responsible for infections in patients with sepsis	10
6.	Table S5: Subset information for loading plot & network node	11
7.	Table S6: Properties of pediatric sepsis and healthy control network	12
8.	Figure S1: Flow cytometry gating strategy for $CD45RA^{-}17A^{+}CD4^{+}$ T cells and $CD45RA^{-}CX3CR1^{+}CTLA4^{+}CD4^{+}$ T cells	13
9.	Figure S2: Flow cytometry gating strategy for $CD15^{+}CD14^{+}$ monocytes and $Ki67^{+}$ B cells	14
10.	Figure S3 Supervised bivariate gating validation of unsupervised FlowSOM-derived cell cluster frequencies (mass cytometry).	15
11.	Figure S4: Stratified analysis by age of immune cell subsets increased in paediatric sepsis.	16
12.	Figure S5: Stratified analysis by age of immune cell subsets decreased in paediatric sepsis.	17
13.	Figure S6: Fluorescence-based flow cytometry validation of mass cytometry identified paediatric sepsis-enriched immune cell subsets.	18
14.	Figure S7: Area under the Receiver operating characteristic analysis of the validation cohort for the diagnosis of sepsis, sepsis shock, severe sepsis and for the prediction of mortality.	19

Detailed Methodology

Patients

This study included 39 consecutive children with sepsis admitted to the paediatric intensive care unit (PICU) and 19 healthy age-matched children. Sepsis was defined as an acute rise in the pediatric sequential organ failure assessment (pSOFA) score≥2 points in the setting of proven or suspected infection[1]. All subjects also fulfilled the International Pediatric Sepsis Consensus Conference definition for sepsis[2]. Patients with known immunodeficiency or on immunosuppressants were excluded. Sepsis management in the unit was based on the Surviving Sepsis Campaign recommendations[3, 4]. Ethical approval from the SingHealth Centralised Institutional Review Board was obtained for this study (CIRB ref. no. 2017-3076 and 2015-2231). Healthy children were recruited among those undergoing elective surgeries with blood obtained at the time of intravenous cannulation prior to the induction of anaesthesia, or post-operatively if the former was not available (CIRB ref no. 2019-2961 and 2015-2231).

Data extraction

Clinical data for enrolled subjects, including demographic, laboratory, source of infection and outcome data were collected (Table 1 and S3). Clinical severity scores including the PIM-3 and PELOD-2 were calculated on PICU admission.

Cell isolation

Blood samples were collected in ethylenediaminetetraacetic acid (EDTA) tubes within 48 hours of sepsis diagnosis. Peripheral blood mononuclear cells (PBMCs) were isolated by density centrifugation using Ficoll-Paque PLUS (GE Healthcare, UK) and subsequently cryopreserved in fetal calf serum (FCS, Gibco, USA) with 10% (v/v) dimethyl sulfoxide (DMSO, Sigma–Aldrich, UK).

Mass Cytometry

Cryopreserved PBMCs were thawed in Roswell Park Memorial Institute 1640 (RPMI) medium supplemented with 10% (v/v) human serum (Corning, USA) and 1× (v/v) penicillin-streptomycin-glutamine (Gibco, USA). Cells were then resuspended in the same medium and rested for 30 minutes at 37ºC. Subsequently, the cells were harvested and stimulated with phorbol 12-myristate 13 acetate (PMA) at 150 ng/ml and ionomycin at 250 ng/ml. PMA and ionomycin (both from Sigma-Aldrich, UK) stimulation was done for 5 hours. PMA-ionomycin bypasses T-cell receptor activation to induce cytokine production enabling it to be detected - the cells intrinsic function/phenotype based on their clinical condition (sepsis vs healthy) is not differentially affected by stimulation [5-8]. Brefeldin A and monensin (eBioscience) were added during the last 3 hours of the incubation for blockade of protein transport.

The cells were processed using the standardized EPIC staining protocol as described previously.[6] In brief, PBMCs were washed once with cell staining buffer (CSB) (phosphate buffered saline [PBS] with 4% FCS, 2 mM EDTA, 0.05% sodium azide) and centrifuged at 524 ×*g* for 6 min at 4ºC. The supernatant was decanted and cells were stained with cisplatin viability stain (PBS with 10 µM cisplatin) (DVS Sciences, USA) for 5 min on ice. PBMCs were then washed and stained with Fluorescein isothiocyanate (FITC) anti-human TCR ɣ/δ (Invitrogen, USA) at 5 µl and a quadruplet barcode system comprising of CD45 antibodies conjugated with Y-89, Cd-106, Cd-113 or Sn-115.[9] After incubation on ice for 20 min, PBMCs were washed three times before they were combined and pelleted in preparation for surface staining with the antibody panel (Table S1). PBMCs were first stained with lanthanide-conjugated surface marker antibodies in room temperature for 15 min in a final reaction volume of 180 µl. After washing twice (initially with CSB and then with 1× PBS), PBMCs were fixed and permeabilized in 1 mL of fixation/permeabilization buffer (eBioscience, USA) for 45 min on

ice. PBMCs were then washed twice with permeabilization wash buffer (eBioscience, USA) and centrifuged at 840 ×*g* for 6 min. After decanting the supernatant, PBMCs were stained with lanthanide-conjugated intracellular marker antibodies on ice for 45 min in a final reaction volume of 180 µl. PBMCs were subsequently washed once with permeabilization wash buffer and resuspended in 1× PBS with 1.6% paraformaldehyde (PFA) for 1 day at 4ºC prior to data acquisition.

On the day of data acquisition, the cells were pelleted and stained with 500 µL DNA intercalator (DVS Sciences, USA) diluted in 1.6% PFA/1× PBS for 20 min, RT. The cells were then washed twice with CSB and twice with cell acquisition solution (CAS) (Standard BioTools, USA). The pelleted cells were resuspended to a density of 10^6/mL in UltraPure DNase/RNase-Free Distilled Water with 10% (v/ v) EQTM Six Element Calibration Beads (Standard BioTools, USA) in accordance to the manufacturer's instructions. Data acquisition was then performed using a XT mass cytometer (Standard BioTools, USA).

Processing of Data Output from XT Mass Cytometer

The XT-generated output files were normalized using EQTM Six Element Calibration Beads.[10] The live single cell events and singlets were gated via 2 steps: first by identifying singlets via a bivariate plot of DNA intercalator versus event length, and next by detecting singlets that are negative for cisplatin as previously described.[9] De-barcoding was carried out using a bivariate gating strategy in FlowJo (Version 10.7.1, Becton, Dickinson & Company, USA) and exported for unsupervised analysis.

Clustering

To identify cell populations in an unsupervised manner, cytometry analysis using self-organising maps (FlowSOM) clustering[11] using a 10×10 grid size was applied after random down-sampling to 10,000 cell events per subject as previously described[6]. All clustering and dimensionality reduction operations were preceded by hyperbolic arcsine transformation with a scale factor of 5 (asinh5). Cells were clustered using the FlowSOM algorithm into 100 nodes with subsequent merging based on phenotypic similarity into 47 unique cell subsets. 46 subsets were obtained after excluding 1 cluster composed of a mixed cell population from our analysis (Table S5). Protein expression patterns of clusters were examined using dendrogram heat maps constructed using the 'heatmaply' R package. Supervised bivariate gating was done to validate the unsupervised FlowSOM-derived cell cluster frequencies (Figure S1-2).

Dimensionality reduction

Non-linear dimensionality reduction was performed using t-Distributed Stochastic Neighbour Embedding (tSNE)[12] 12 to visualize multi-dimensional expression landscapes in two dimensions (2D). 'Relatedness' among different clusters was visualised after embedding FlowSOM clustering information onto the 2D tSNE plots.

Network analysis

The proportion of nodes (Immune cell subsets) was calculated for every patient, and the correlation between nodes was calculated for healthy control and pediatric sepsis (Table S6). To construct the network, nodes were connected if they had an absolute correlation coefficient >0.6. The network was visualized and analyzed using the igraph R package. The correlation network was plotted using a force-directed Fruchterman-Reingold graph layout.[13]

Flow cytometry validation

Significantly increased subsets identified on mass cytometry were validated using flow cytometry in a cohort of 11 paediatric sepsis and 11 healthy controls (age-matched). Two flow cytometry antibody panels (Table S2) were designed for the validation. The thawing, stimulation and staining protocol mirrored that of the mass cytometry protocol described above, with the exception that Live/Dead blue dye (Invitrogen) was used for 15 min at room

temperature in PBS. Stained samples were then analyzed using LSR Fortessa™ flow cytometer (BD Bioscience). Verification of FlowSOM clustering frequency (expressed as a percentage of $CD45^+$ PBMCs) was performed with bivariate supervised gating in FlowJo (Version 10.7.1, BD, USA) (Figure S3).

Statistical analysis
Cell subset frequencies were plotted as median with interquartile range (IQR). Chi-squared/Fisher's exact and Mann–Whitney U and tests were used to compare groups where appropriate. To account for type I error, Bonferroni correction was used when comparing the FlowSOM cell clusters obtained from the mass cytometry data between sepsis and control groups. To determine the effect of age, stratified analysis by age groups ≤1year vs >1year was performed as subgroup analysis (Figure S4 and S5). Correlation between variables was calculated either with Pearson (r) or Spearman (r_s) correlation coefficients (Figure 2C). Principal component analysis (PCA) was used to extract and visualise the dominant patterns in the matrix (Table 2, Figure 1C). Significantly increased cell subsets in pediatric sepsis were considered for inclusion in a model to differentiate sepsis from healthy, and a model to predict mortality. The discriminative ability and performance of each significantly increased/decreased cell subset (Figure 3) and the combined model was assessed by calculating the Area Under the Curve from final Receiver Operating Characteristics Curve (AUROC), sensitivity and specificity. AUROC was similarly calculated for clinical scores and routine laboratory markers (e.g., procalcitonin and lactate). Analyses were performed using SPSS, version 23.0 (IBM Corp., NY, USA) and GraphPad Prism V.7 (GraphPad Software, Inc., CA, USA) with statistical significance set at $p < 0.05$.

Table S1. Mass Cytometry panel antibodies conjugated metal, clone and vendor information

Metal	Antibody	Clone	Vendor
89	CD45A	HI30	Fluidigm
106	CD45B	HI30	Fluidigm
110	CD19	HIB19	Biolegend
111	CD45RA	HI100	Biolegend
112	CD14	Tuk4	Invitrogen
113	CD45C	HI30	Fluidigm
114	CD56	NCAM16.2	BD
115	CD45D	HI30	Biolegend
116	CD8	SK1	Biolegend
139	HLA-DR	L243	Biolegend
141	CD244	C1.7	Biolegend
142	CD107A	H4A3	Biolegend
143	CD3	UCHT1	Biolegend
144	IL6	MQ2-13A5	Biolegend
145	IL4	8D4-8	Biolegend
146	TCRGD_FITC	FIT-22	Biolegend
147	PD-1	EH12.2H7	Biolegend
148	CD4	SK3	Biolegend
149	IL-2	MQ1-17H12	Biolegend
150	CCR4	L291H4	Biolegend
151	GATA3	TWAJ	eBioscience
152	TNF-a	Mab11	Biolegend
153	CD25	2A3	bdbiosciences
154	CD15	H198	Biolegend
155	CD152	BNI3	BD
156	CD28	CD28.2	Biolegend
157	CXCR5	RF8B2	BD
158	CCR7	G043H7	Biolegend
159	CXCR3	G025H7	Biolegend
160	CD161	HP-3G10	Biolegend
161	CCR2	K036C2	Biolegend
162	CD160	BY55	Biolegend
163	TBX21/T-bet	4B10	BioXell
164	CX3CR1	K0124E1	Biolegend
165	FoxP3	PCH101	eBioscience
166	Ki67	20Raj1	eBioscience
167	TCRa7.2	3C10	Biolegend
168	IFN-ɣ	B27	Biolegend
169	IL-17A	BL168	Biolegend
170	IL-8	BH0814	Biolegend
171	TIGIT	MBSA43	Invitrogen
172	IL-1B	JK1B-1	Biolegend
173	GranzymeB	ab103159	Abcam
174	CD33	WM53	Biolegend

175	MRGX2 (MasR)	K125H4	Biolegend
176	iCOS	C398.4A	Biolegend
191/193	DNA		
195	Live/Dead		
209	CD16	3G8	Fluidigm

Table S2. Flow Cytometry Validation Panel

	Marker	Clone	Cat#	Vendor
Panel 1	CD4	SK3	564306	BD
	CD3	UCHT1	563546	BD
	IL-17A	BL168	512328	Biolegend
	TIGIT	A15153G	372712	Biolegend
	CD45RA	HI100	304130	Biolegend
	CD8	SK1	347313	BD
	FoxP3	PCH101	25-4776-42	eBioscience
	CTLA4/CD152	BNI3	555854	BD
	CXCR3	G025H7	353736	Biolegend
	CX3CR1	K0124E1	355704	Biolegend
	CD25	2A3	557753	BD
	CCR7	G043H7	353244	Biolegend
	CD160	BY55	341208	Biolegend
Panel 2	CD3	UCHT1	564306	BD
	IgD	IA6-2	348242	Biolegend
	CD38	HIT2	303528	Biolegend
	CD19	HIB19	302244	Biolegend
	CD27	O323	302824	Biolegend
	CXCR3	G025H7	353713	Biolegend
	CD138	MI15	356508	Biolegend
	Ki67	20Raj1	25-5699-42	eBioscience
	CX3CR1	K0124E1	355704	Biolegend
	CD15	H198	301920	Biolegend
	CD14	M5E2	301808	Biolegend

Table S3. Demographic and clinical characteristics of sepsis survivors and non-survivors

	Survivors (n=51)	Non-survivors (n=8)
**Demographics**		
Age(years)	5.0 (2.0, 10.5)	9.1 (2.7, 13.7)
Male gender,n(%)	32 (62.7)	3 (37.5)
**Laboratory examinations**		
White blood count, 10^9 /L	9.5 (5.4, 20.1)	12.8 (5.3, 35.2)
C-reactive protein, mg/L	119.0 (32.1, 190.7)	140.1 (26.2, 328.6)
Procalcitonin, ng/mL	7.1 (0.9, 62.0)	4.2 (1.0, 63.7)
Lactate, mmol/L	1.6 (1.0, 3.1)	1.8 (1.2, 5.5)
**Severity of disease***		
pSOFA	8.0 (4.5, 10.5)	12.0 (8.0, 13.5)
PIM-3	3.3 (1.6, 5.8)	8.0 (3.1, 10.3)
PELOD-2	6 (3, 8)	8.5 (5.5, 12)

*Scored at PICU admission. Data are expressed as median (interquartile range) unless otherwise indicated. PELOD-2: pediatric logistic organ dysfunction 2 score; PIM3: pediatric index of mortality 3; pSOFA: pediatric sequential organ failure assessment; PICU: pediatric intensive care unit.

Table S4. Pathogens responsible for infections in septic patients at admission in the cohort

Infective agents	Number
**Bacteria**	
Streptococcus pneumoniae	4
Streptococcus agalactiae	3
Streptococcus pyogenes	3
Staphylococcus aureaus	4
Mycoplasma pneumoniae	2
Hemophilus infleunza	1
Enterococcus faecium	1
Pseudomonas aeruginosa	1
Escherichia coli	1
Serratia marcescens	1
**Virus**	
Influenza A, B virus	11
Respiratory syncytial virus	6
Adenovirus	5
Parainfluenza 1, 2, 3	2
Metapneumovirus	1
Human coronavirus	1
**None**	19

NB: Patients may have more than one infective agent identified.

Table S5. Subset information for loading plot and network node

Subsets		Subset number
CD4	$CD45RA^-CX3CR1^+CTLA4^+CD4^+$	2
	$CD45RA^-IL17A^+CD4^+$	4
	$IL8^+$ naïve CD4	7
	Temra CD4	11
	$CD160^+CX3CR1^+CXCR3^+CCR4^+$ effector memory CD4	13
	Central memory CD4	16
	$CX3CR1^+CXCR3^+$ naïve CD4	18
	Effector memory CD4	22
	$FOXP3^+CD25^+CD152^+$ naïve Treg	23
	$FOXP3^+CD25^+CD152^+PD1^+TIGIT^+CCR4^+$ Treg	24
	$IL2^+CD152^+$ central memory CD4	25
	$IL4^+$ naïve CD4	26
	$IL4^+TIGIT^+CX3CR1^+CXCR3^+CCR4^+$ naïve CD4	27
	naïve CD4	32
	$PD1^+CD152^+CD161^+$ effector memory CD4	34
	$PD1^+CD152^+Ki67^+$ central memory CD4	35
	$PD1^+CD152^+TIGIT^+$ effector memory CD4	36
	$TNF\alpha^+IL2^+$ effector memory CD4	45
	$TNF\alpha^+IL2^+IFN\gamma^+$ effector memory CD4	46
CD8	$Tbet^+CD244^+$ temra CD8	9
	$IL8^+$ naïve CD8	28
	naïve CD8	33
	$Tbet^+CD244^+GB^+IFN\gamma^+IL1\beta^+$ temra CD8	37
	$Tbet^+CD244^+GB^+IL1\beta^+$ temra CD8	38
	$CX3CR1^+CXCR3^+CD160^+$ naïve CD8	20
	$Tbet^+GB^+IFN\gamma^+IL6^+$ effector memory CD8	41
	$Tbet^+GB^+IFN\gamma^+IL6^+CD244^+PD1^+$ effector memory CD8	42
	$Tbet^+GB^+IFN\gamma^+IL6^+IL1\beta^+PD1^+CD152^+Ki67^+$ effector memory CD8	43
	Temra CD8	44
B	$Ki67^+$ B	3
	B	12
	$CX3CR1^+CXCR3^+CCR4^+CD160^+$ B	19
NK	$GATA3^+CD56^{++}$	10
	$CD56^+$	14
Myeloid	$CD15^+CD14^+$ monocyte	1
	$Lin^-HLADR^+IL8^+$	8
	Classical monocyte	15
	$CX3CR1^+CXCR3^+$ monocyte	17
	Lin^-HLADR^+	30
Double negative T	γδT	6
	Double negative T	21
	Mucosal associated invariant T (MAIT)	31
	$Tbet^+GB^+CX3CR1^+CXCR3^+$ γδT	39
	$Tbet^+GB^+IFN\gamma^+$ γδT	40
$Lineage^-$	Type 2 innate lymphoid cells (ILC2)	5
	Lin^-	29

Lin^-: lineage negative denotes $CD3^-CD4^-CD8^-CD14^-CD19^-CD56^-$, GB: granzyme B.

Table S6. Properties of pediatric sepsis and healthy control network

Network property	Pediatric sepsis	Healthy control
Network density	0.040	0.103
Network centralization	0.069	0.220
Modularity score	0.242	0.002
Negatively correlated edges	0	8
Positively correlated edges	41	99

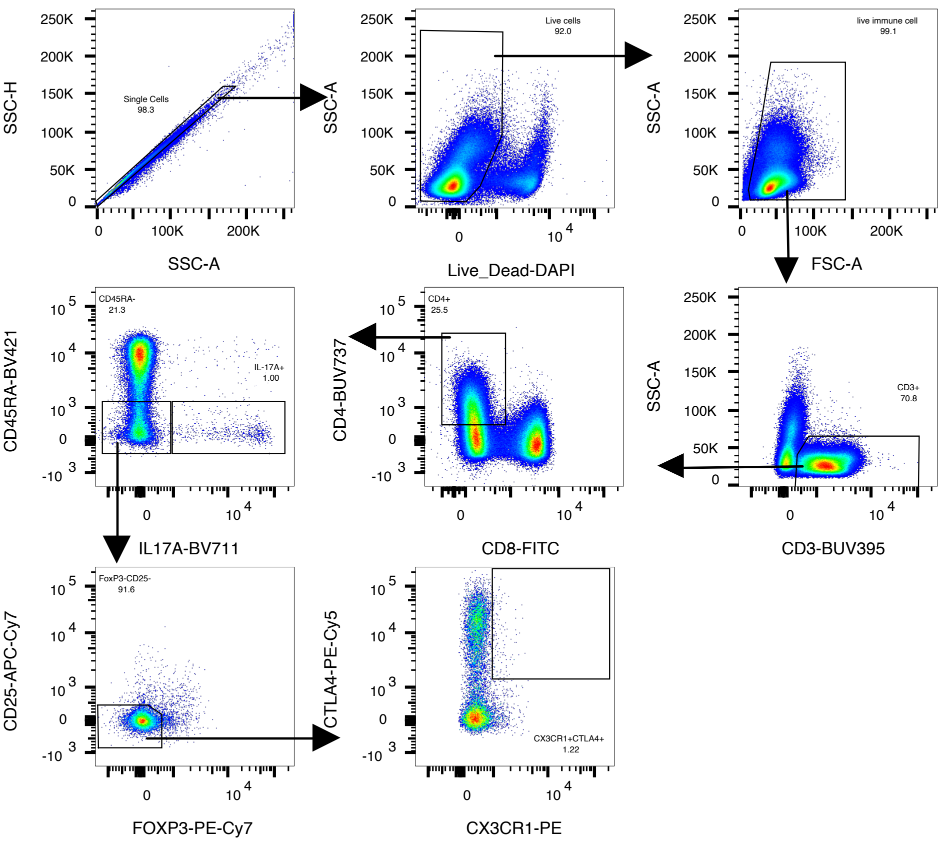

Figure S1: Flow cytometry gating strategy for CD45RA$^-$17A$^+$CD4$^+$ T cells and CD45RA$^-$CX3CR1$^+$CTLA4$^+$CD4$^+$ T cells.

Stimulated PBMCs (with PMA and ionomycin) were studied. Antibody panel 1 was used (Table S2).

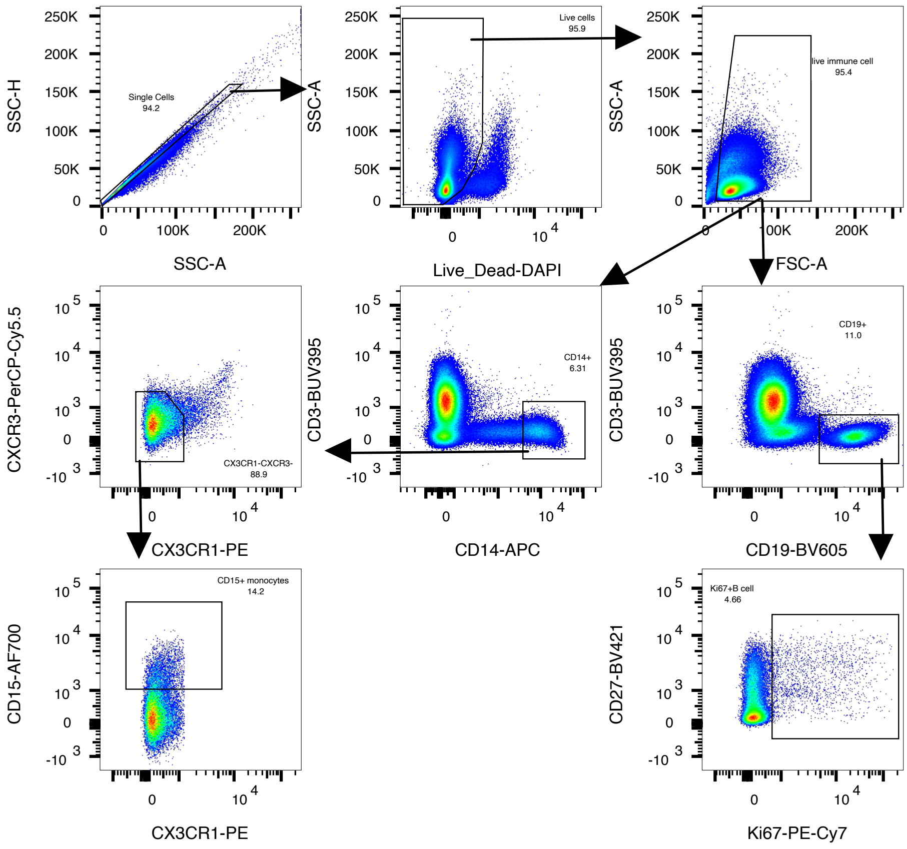

Figure S2: Flow cytometry gating strategy for CD15^{+}CD14^{+} monocytes and Ki67^{+} B cells
Stimulated PBMCs (with PMA and ionomycin) were studied. Antibody panel 2 was used (Table S2).

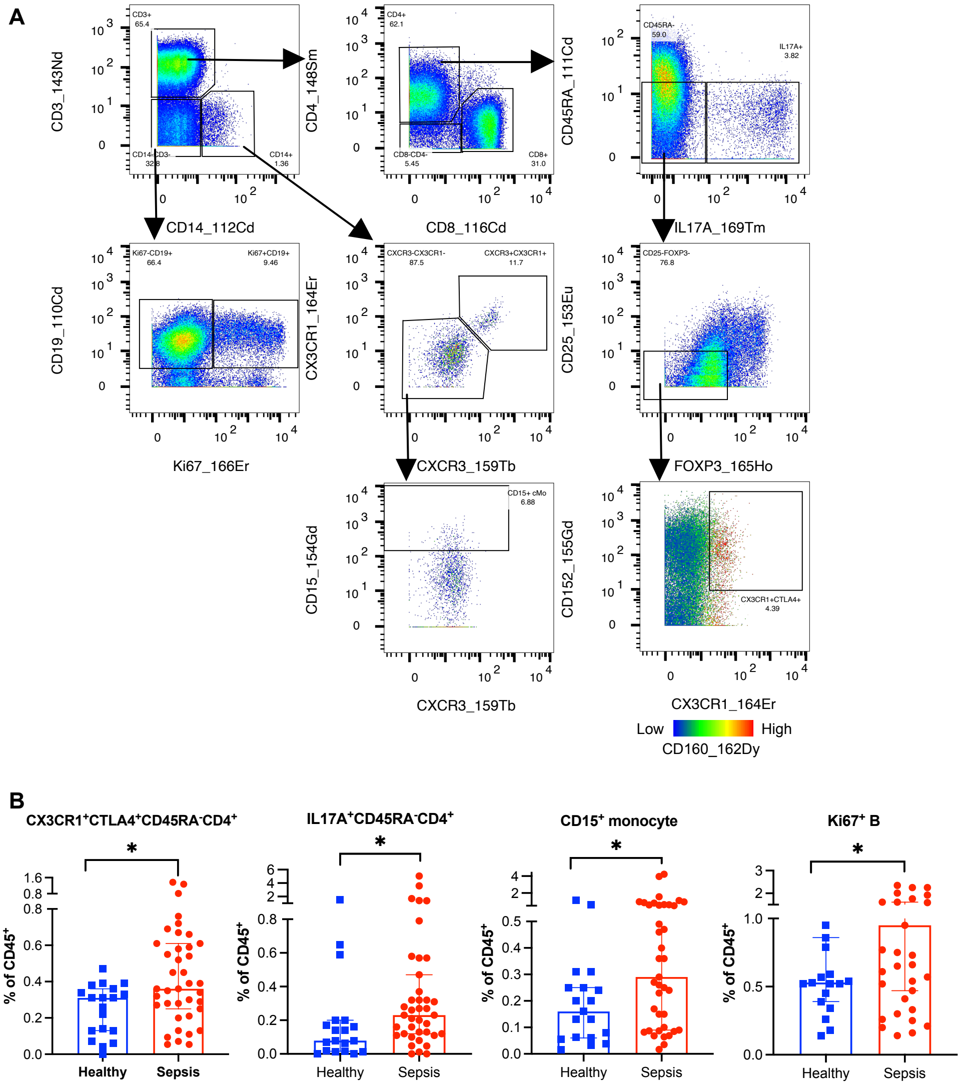

Figure S3 Supervised bivariate gating validation of unsupervised FlowSOM-derived cell cluster frequencies (mass cytometry).

A, Gating strategy for CD15$^+$CD14$^+$ monocytes, CD45RA$^-$17A$^+$CD4$^+$ T cells, CD45RA$^-$CX3CR1$^+$CTLA4$^+$CD4$^+$ T cells and Ki67$^+$ B cells. B, Differences in the bivariate gating derived frequencies of the 4 immune cell subsets between paediatric sepsis and in health. Median and IQR are shown. Statistical testing with Mann Whitney U test: *p<0.05, **p<0.01, ***p<0.001, ****p<0.0001.

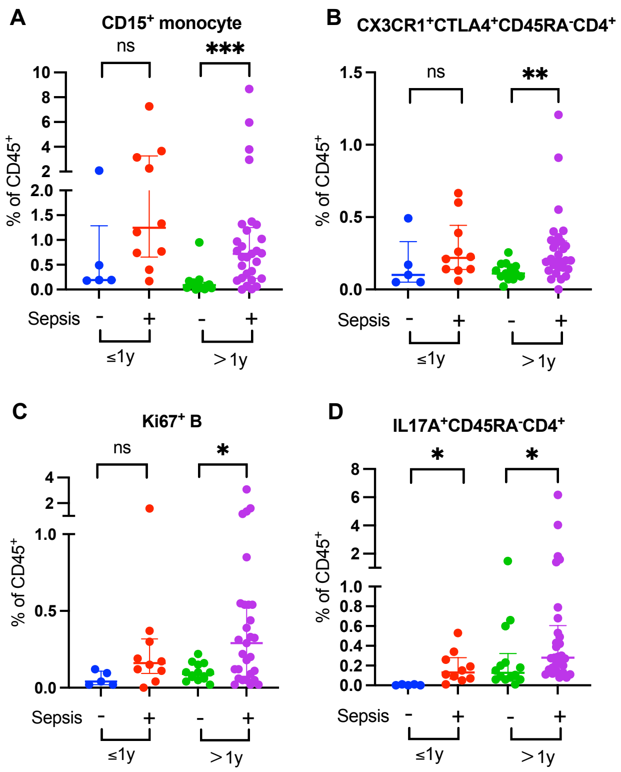

E

	Sepsis group		***P* value**
	**Age under 1y (n=10)**	**Age above 1y (n=29)**	
**SOFA scores**	10.5 (4.5-12.8)	8 (6-11)	0.572
**PELOD-2**	7.5 (3-11.3)	7 (5-8)	0.756
**PIM-3**	5.4 (4.2-7.6)	3.1 (1.5-4.5)	0.037
**Mortality in PICU (n, %)**	1 (10%)	5 (17.2%)	>0.999#

Figure S4: Stratified analysis by age of immune cell subsets increased in paediatric sepsis. A-C, $CD15^+CD14^+$ monocytes, $CD45RA^-CX3CR1^+CTLA4^+CD4^+$ T cells and $Ki67^+$ B cells were only significantly increased in those above 1 year old in paediatric sepsis. D, $CD45RA^-17A^+CD4^+$ T cells were significantly elevated in both age groups. Median and IQR are shown. Statistical testing with Mann Whitney U test: *p<0.05, **p<0.01, ***p<0.001, ****p<0.0001. E, Clinical characteristics of the sepsis group by age. There are no differences in SOFA, PELOD-2 and mortality between the two age groups. The PIM-3 in the ≤ 1 year old is significantly higher than the > 1 year old (*p<0.05, #: Fisher exact test).

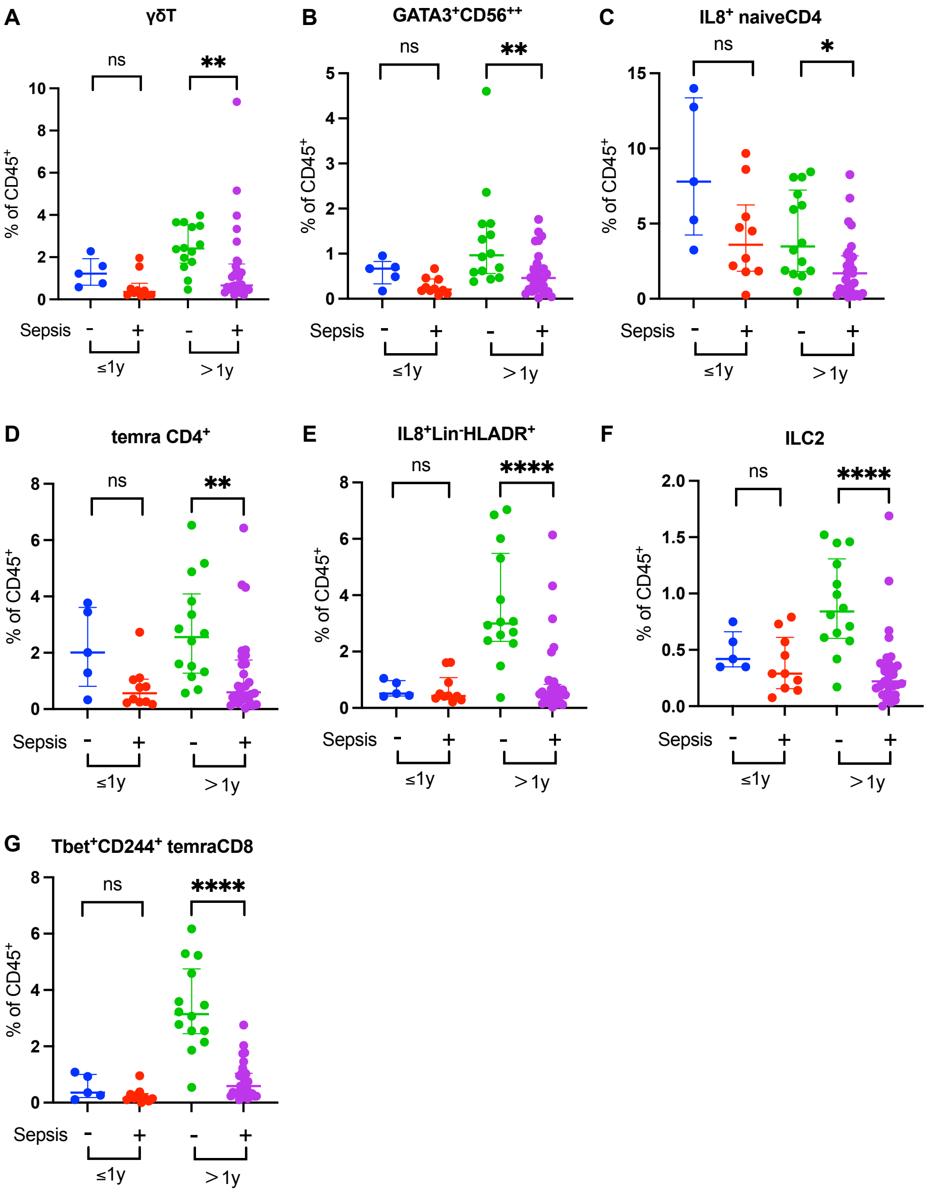

Figure S5 Stratified analysis by age of immune cell subsets decreased in paediatric sepsis. A-G, The 7 subsets were only significantly decreased in those above 1 year old. Median and IQR are shown. Statistical testing with Mann Whitney U test: *p<0.05, **p<0.01, ***p<0.001, ****p<0.0001.

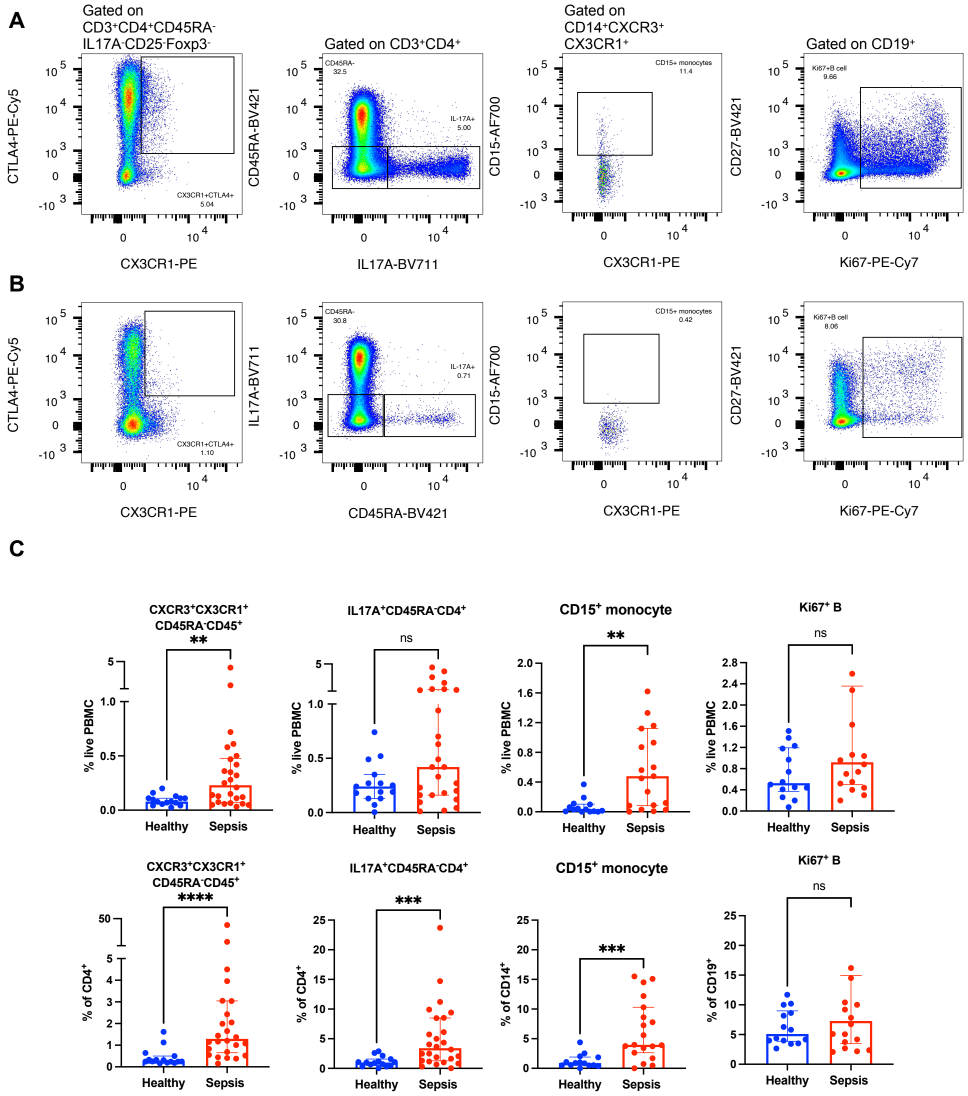

Figure S6: Fluorescence-based flow cytometry validation of mass cytometry identified paediatric sepsis-enriched immune cell subsets.

A, Representative gating strategy on a paediatric patient with sepsis. B, Representative gating on a healthy donor. C, Comparison of 4 immune subsets between paediatric sepsis and in health. Their frequencies are depicted as a percentage of total live PBMC and as a percentage of its major lineage, $CD4^+$, $CD14^+$ or $CD19^+$ (n = 11, paediatric sepsis and n = 11, healthy donors). The increase in 3 of the immune cell subsets in paediatric sepsis: $CD15^+CD14^+$ monocytes, $CD45RA^-17A^+CD4^+$ T cells, $CD45RA^-CX3CR1^+CTLA4^+CD4^+$ T cells are validated with flow cytometry. Median and IQR are shown. Stimulated PBMCs (with PMA and ionomycin) were studied. Statistical testing with Mann Whitney U test: *p<0.05, **p<0.01, ***p<0.001, ****p<0.0001.

A

	Sepsis	Septic shock	Severe sepsis	Mortality
CD15+CD14+ monocytes (% PBMC)	0.79*	0.83*	0.88*	0.56
CX3CR1$^+$CTLA4$^+$CD4$^+$ T cells (% PBMC)	0.74*	0.75*	0.64	0.45
CD45RA$^-$17A$^+$CD4$^+$ T cells (% PBMC)	0.55	0.59	0.63	0.53
Ki67$^+$ B cells (% PBMC)	0.67	0.63	0.55	0.56
CD15+CD14+ monocytes (% CD14+)	0.78*	0.79*	0.86*	0.48
CX3CR1$^+$CTLA4$^+$CD4$^+$ T cells (% CD4+)	0.90*	0.93*	0.79*	0.73
CD45RA$^-$17A$^+$CD4$^+$ T cells (% CD4+)	0.76*	0.80*	0.79*	0.60
Ki67$^+$ B cells (% CD19+)	0.67	0.66	0.56	0.46
Four cell subset (% PBMC)	0.89*	0.82*	0.81*	0.68
Three cell subset (% PBMC)	0.91*	0.83*	0.80*	0.72
Four cell subset (% lineage)	0.95*	0.90*	0.94*	0.94*
Three cell subset (% lineage)	0.94*	0.93*	0.94*	0.80
Four cell subset (% PBMC, adjusted for age)	0.87*	0.89*	0.82*	0.92*
Three cell subset (% PBMC, adjusted for age)	0.86*	0.89*	0.80*	0.92*
Four cell subset (% lineage, adjusted for age)	0.94*	0.95*	0.95*	1.00*
Three cell subset (% lineage, adjusted for age)	0.94*	0.94*	0.94*	0.94*

Figure S7: Area under the Receiver operating characteristic analysis of the validation cohort for the diagnosis of sepsis, sepsis shock, severe sepsis and for the prediction of mortality.

AUROC values derived from flow cytometry data from the validation cohort (n = 35). *Indicates that the 95% confidence intervals do not cross 0.5.

References:

1. Matics TJ, Sanchez-Pinto LN: Adaptation and Validation of a Pediatric Sequential Organ Failure Assessment Score and Evaluation of the Sepsis-3 Definitions in Critically Ill Children. *JAMA Pediatrics* 2017, 171(10):e172352-e172352.
2. Goldstein B, Giroir B, Randolph A: International pediatric sepsis consensus conference: definitions for sepsis and organ dysfunction in pediatrics. *Pediatric critical care medicine : a journal of the Society of Critical Care Medicine and the World Federation of Pediatric Intensive and Critical Care Societies* 2005, 6(1):2-8.
3. Rhodes A, Evans LE, Alhazzani W, Levy MM, Antonelli M, Ferrer R, Kumar A, Sevransky JE, Sprung CL, Nunnally ME *et al*: Surviving Sepsis Campaign: International Guidelines for Management of Sepsis and Septic Shock: 2016. *Intensive Care Med* 2017, 43(3):304-377.
4. Weiss SL, Peters MJ, Alhazzani W, Agus MSD, Flori HR, Inwald DP, Nadel S, Schlapbach LJ, Tasker RC, Argent AC *et al*: Surviving Sepsis Campaign International Guidelines for the Management of Septic Shock and Sepsis-Associated Organ Dysfunction in Children. *Pediatric Critical Care Medicine* 2020, 21(2):e52-e106.
5. Yeo JG, Leong JY, Tay SH, Nadua KD, Anderson DE, Lim AJM, Ng XW, Poh SL, Guo D, Yaung KN *et al*: A Virus-Specific Immune Rheostat in the Immunome of Patients Recovering From Mild COVID-19. *Frontiers in immunology* 2021, 12:674279.
6. Yeo JG, Wasser M, Kumar P, Pan L, Poh SL, Ally F, Arkachaisri T, Lim AJM, Leong JY, Lai L *et al*: The Extended Polydimensional Immunome Characterization (EPIC) web-based reference and discovery tool for cytometry data. *Nat Biotechnol* 2020, 38(6):679-684.
7. Kumar P, Shih DCW, Lim A, Paleja B, Ling S, Li Yun L, Li Poh S, Ngoh A, Arkachaisri T, Yeo JG *et al*: Pro-inflammatory, IL-17 pathways dominate the architecture of the immunome in pediatric refractory epilepsy. *JCI Insight* 2019, 5.
8. Lin D, Gupta S, Maecker HT: Intracellular Cytokine Staining on PBMCs Using CyTOF™ Mass Cytometry. *Bio Protoc* 2015, 5(1).
9. Lai L, Ong R, Li J, Albani S: A CD45-based barcoding approach to multiplex mass-cytometry (CyTOF). *Cytometry A* 2015, 87(4):369-374.
10. Finck R, Simonds EF, Jager A, Krishnaswamy S, Sachs K, Fantl W, Pe'er D, Nolan GP, Bendall SC: Normalization of mass cytometry data with bead standards. *Cytometry A* 2013, 83(5):483-494.
11. Van Gassen S, Callebaut B, Van Helden MJ, Lambrecht BN, Demeester P, Dhaene T, Saeys Y: FlowSOM: Using self-organizing maps for visualization and interpretation of cytometry data. *Cytometry A* 2015, 87(7):636-645.
12. Van der Maaten L, Hinton G: Visualizing data using t-SNE. *Journal of machine learning research* 2008, 9(11).
13. Kumar P, Shih DCW, Lim A, Paleja B, Ling S, Li Yun L, Li Poh S, Ngoh A, Arkachaisri T, Yeo JG *et al*: Pro-inflammatory, IL-17 pathways dominate the architecture of the immunome in pediatric refractory epilepsy. *JCI Insight* 2019, 5(8).
